# Material Nondestructive Investigations Reveal the Hidden Secrets of Two Saxon Quarter Thalers Issued in 1544—A Case Study

**DOI:** 10.3390/ma19071325

**Published:** 2026-03-26

**Authors:** Marzena Grochowska-Jasnos, Emanoil Pripon, Lucian Barbu Tudoran, Nicoleta Ignat, Gheorghe Borodi, Ioan Petean

**Affiliations:** 1 Museum of Archaeology and History in Glogow, Brama Brzostowska 1, 67-200 Glogow, Poland; marzena.grochowska@gmail.com; 2 Zalau County Museum of History and Art, 9 Unirii Street, 450042 Zalau, Romania; emanoilpripon@gmail.com; 3 Faculty of Biology and Geology, Babes-Bolyai University, 44 Gheorghe Bilaşcu Street, 400015 Cluj-Napoca, Romania; lucian.barbu@ubbcluj.ro; 4 National Institute for Research and Development of Isotopic and Molecular Technologies, 65-103 Donath Street, 400293 Cluj-Napoca, Romania; borodi@itim-cj.ro; 5 Faculty of Chemistry and Chemical Engineering, Babes-Bolyai University, 11 Arany Janos Street, 400028 Cluj-Napoca, Romania; nicoleta.cotolan@ubbcluj.ro

**Keywords:** Saxony, quarter thaler, nondestructive investigation, XRD, SEM-RDS, XRF

## Abstract

Saxony was ruled by two cousins in 1544: John Frederick I (Elector of Saxony) and his cousin Maurice (Duke of Saxony). Both rulers’ names appear on each side of the quarter thalers produced in this year. They were enemies involved in religious wars, although they were both Protestants. Two types of quarter thalers from 1544 occur: a pierced random find from Transylvania (Romania) with four shields on the reverse, heavily worn, and another one with three shields on the obverse side, found in the Głogów Hoard (Poland), which is well preserved. Why did they issue two types in the same year? Was it a matter of silver title or other historical factors? Nondestructive investigation methods were used: XRD revealed the phases within the alloy and patina layer; SEM-EDS revealed the morphological aspects and their elemental compositions, which were correlated with XRF results. The results show that both coins have closer silver amounts, from 91 to 96 wt.%. The EDS results were in good agreement with the XRF results. Lead traces indicated a difference between them: the four-shielded coin is lead-free, while the three-shielded coin has a moderate amount of lead, about 0.5 wt.%. The archeological data evidence that the four-shielded coin issued in 1544 is rarer than the three-shielded one because it was issued during specific historical conditions. Black patina is formed by a mixture rich in copper oxides mixed with silver oxides and Ag_2_S. The presence of silver sulfide in the patina layer confirms that the pierced coin was in prolonged contact with the skin surface. Also, the finest traces of minerals embedded in the patina layer (e.g., quartz, kaolinite, and calcite) suggest that they were embedded in the patina via prolonged exposure to particulate matter. The mineral inclusions in the patina would have been more numerous if they were formed underground. Thus, the pierced four-shielded coin was probably worn as jewelry by nomads, while the three-shielded coin was most likely treasured in a well-preserved hoard.

## 1. Introduction

Archeological artifacts are made of various materials, including wood, metal and stone, and very often comprise precious metals and stones [1,2]. Their detailed investigation is of great interest for archeologists, revealing their complex nature related to the specific manufacturing technologies available during different ages [3,4]. An artifact’s microstructure and elemental composition help elucidate specific debates among historians, sometimes proving or contradicting the circulating theories regarding a specific time or era. Theoretical debates require solid proof based on artifact investigations for their validation beyond any doubt.

Unfortunately, archeological artifacts are priceless in most cases, and investigative sampling of a collection might be very difficult or even impossible. Therefore, nondestructive investigation methods have gained importance, becoming powerful tools of archeological investigation. X-ray fluorescence (XRF) devices are very versatile for revealing the elemental composition of artifacts; having a compact and portable design, these devices allow in situ investigation where an artifact is found or exposed [5,6]. The major drawback of this method is that it reads only from the artifact’s surface and cannot perform deeper sampling of its bulk composition [7]. Moreover, surface contamination on an artifact, such as soil particles or other compounds, is prone to influence XRF readings, which are very sensitive [8,9]. Therefore, the artifact surface must be properly conditioned and cleaned with care for XRF to measure the material’s composition without being affected by cleaning agents [10]. Scanning Electron Microscopy (SEM) allows high-resolution imaging of an artifact’s microstructure, which can be precisely related to its elemental composition through Energy-Dispersive Spectroscopy (EDS) analysis, which measures the elemental composition as well as XRF and facilitates acquisition of elemental distribution maps through backscattered electron (BSE) images [11,12]. The main drawback of this method is the limited artifact size of a few centimeters (e.g., 10–20 cm), and it requires transport to a microscopy laboratory [13]. Thus, bigger artifacts require the delicate removal of small samples before investigation. Removing small parts of the artifacts can be destructive in some cases, but very often, restoration specialists can gently remove some parts for analysis without affecting the artifact’s integrity.

Elemental composition data is important for elucidating the mysteries of an archeological material, but it cannot give precise information regarding the specific phases involved in that material. Several phases, like different minerals or different alloy phases, might occur in the same artifact [14] and require a specific investigation able to identify them with great accuracy. X-ray diffraction (XRD) investigation allows precise identification of the involved minerals and alloy phases, completing the complex physicochemical characterization of the artifact [15,16]. Small artifacts can be directly investigated, while bigger ones require sampling of a small quantity of powder ground off from the unexposed side [17]. Some additional semi-nondestructive methods (e.g., requiring a small amount of powder collected from the artifact), like Fourier Transform Infrared (FTIR) spectroscopy, allow the precise identification of the involved chemical bonds [18,19], and Gas Chromatography coupled with Mass Spectrometry (GC-MS) allows the identification of specific volatile organic compounds emitted by the artifact. For example, such advanced investigations were able to prove the presence of wine in ancient amphorae [20,21].

Coins are metallic artifacts that are suitably sized for completely nondestructive XRD and SEM-EDS investigation, allowing the proper investigation of their phase and elemental composition. Their alloy microstructure depends on their phase diagrams [22] and the technological processes used for their manufacturing [23]. Metallographic microscopy is usually requested in such a situation, but it requires grinding, polishing, and subsequent chemical etching, which would harm artifacts [24,25], thus resulting in a destructive investigation. The elemental map distribution obtained from SEM-EDS investigation can successfully replace metallographic investigation by revealing grain phases colored in specific nuances, depending on local variance, which is one of the main objectives in the current investigation. The challenge lies in the detection of Ag and Cu atoms regarding the Ag-Cu binary system. It is characterized by α phase, pure silver, β phase, pure copper phase, and the eutectic composition containing a fine lamellar dispersion of E = 71.9 wt.% Ag + 28.1 wt.% Cu. Thus, silver hypoeutectic alloys contain α grains mixed with eutectic grains, while hypereutectic alloys contain eutectic grains mixed with β grains. An elemental map superimposed on a BSE image has already delivered consistent insights for metallographic analysis, highlighting both the grain distribution and their morphologic aspect within Dacian thin rich mirrors [26] or Dacian silver bracelets versus Dyrrhachium drachmas [27].

Thalers, as well as half and quarter thalers, typically had a silver fineness of about 875 ‰ during the 16th and 17th centuries, resembling the Joachimsthaler etalon [28], clearly indicating a hypoeutectic alloy. Saxon adhered to this common standard, ensuring strong compatibility with those of neighboring states [29]. This was made possible by the discovery of rich silver deposits in the Ore Mountains at the end of the 15th century. Saxony was ruled by the House of Wettin, which was split into two branches according to the Treaty of Leipzig in 1485: the Albertine, ruling over the Ducal Saxony, and the Ernestine branch, ruling over the Saxony Electorate [30]. Subsequently, it had two rulers: one was the Saxony Duke, and the other one was the Saxony Elector (a very high rank in the Saint German Roman Empire, involved in the emperor’s election) [31]. The political weight wielded by the Saxon rulers was based on their contiguous territory in central Germany. It essentially consisted of three parts: the Landgraviate of Thüringen, the Electorate and Duchy of Saxony, and the Margraviate of Meissen. In addition, the Saxon dukes held the office of Burgrave in Magdeburg and were able to gain influence through an inheritance agreement with the princely counts of Henneberg in Franconia [32]. The silver coinage of sixteenth-century Saxony played a key role in the development of the European monetary system. The emergence of large silver denominations like the Guldengroschen—later called the thaler—represented a key step in the shift from medieval monetary frameworks to early modern currency systems. These developments were closely connected with the rich silver deposits of the Ore Mountains (Erzgebirge) and the intensive mining activity that flourished there from the late fifteenth century onward. Numerous studies in the numismatic literature have examined the historical development of Saxon coinage, the monetary reforms of the Wettin rulers, and the technological organization of the mints operating in the mining districts of Schneeberg, Annaberg, Freiberg, and Buchholz [32,33,34,35,36,37].

We can now turn to our central figures—Elector John Frederick I, representative of the Ernestine line, and Duke Maurice, representative of the Albertine line, who was 18 years younger. Relations between the cousins had already been tense. The literature reveals that Maurice and John Frederick I were, in fact, enemies. Maurice distanced himself from the Schmalkaldic League, even though its leader was his father-in-law, Landgrave Philip of Hesse. In the next few years, he even allied himself with the Emperor against the Schmalkaldic League. One of the reasons for this was his political rivalry with John Frederick, who held the title of elector, which was also reflected in disputes over income and territorial sovereignty, including the conflict over Wurzen in 1542. (Wurzen was property of the bishop of Meissen, whose see was under the joint protection of electoral and ducal Saxony.) Duke Maurice also disliked the common coinage. Among the denominations introduced during this period were fractional issues of the Guldengroschen, including the quarter thaler (¼ Guldengroschen). These coins circulated widely within the Holy Roman Empire and beyond, reflecting both regional economic activity and the broader integration of Central European monetary systems. Their typology, iconography, and mint organization have been discussed in detail in numismatic scholarship, which has established the main chronological and historical framework for their production. Their disputes affect coin issues, implying splitting access to Saxony’s rich silver ore deposits and coin manufacturing in specific mints.

At the same time, archaeometric investigations of historical coins have increasingly contributed to the understanding of minting technology, alloy composition, and post-depositional processes affecting metallic artefacts. Analytical techniques such as X-ray fluorescence (XRF) and SEM with EDS make it possible to examine the elemental composition of historical silver alloys and to compare measured values with the standards prescribed in historical mint ordinances.

The present study focuses on two Saxon quarter thalers dated to 1544 that represent two slightly different iconographic variants associated with the coinage of Elector John Frederick I and Duke Maurice of Saxony. Given that only two specimens were examined, the work is conceived mainly as a case study that integrates numismatic interpretation with the physicochemical examination of the coin metal and its corrosion products. The aim is not to reconstruct the entire minting system of mid-sixteenth-century Saxony, which has already been extensively discussed in the literature, but rather to illustrate how archaeometric analysis can complement traditional numismatic research.

By comparing the analytical results obtained from these two coins with the standards described in historical sources and numismatic research, the study aims to assess how closely the measured composition aligns with expected alloy parameters and to examine possible technological and post-depositional factors that influence the observed results.

A three-shielded Saxon quarter thaler from 1544 in optimal preservation condition was found in the Glogow Hoard, Poland [38], which can be used as a reference sample for the Transylvanian random find for nondestructive investigation of alloy composition and microstructure. The advanced nondestructive investigation of a random find quarter thaler can be effectuated by XRD, revealing the alloy phases, and SEM coupled with EDS spectroscopy, revealing the alloy composition (e.g., silver title) and the element distribution regarding the grain’s microstructure. All these analyses are nondestructive, preserving the actual shape of the coin. The oxidized outermost layers beside the worn areas of the coin surface form a complex patina that stores physicochemical interactions of the coin during circulation and resting periods. Nondestructive investigations can identify corrosion products and how they were formed through XRD and mineralogical optical microscopy. These investigations may also detect mineral particles embedded into the oxide matrix due to the interaction with resting soil [39,40] or perhaps with environmental particulate matter from the atmosphere [41,42], which may have become incorporated into the patina layer either during circulation or later as a result of weathering. The morphological aspects observed with SEM microscopy allow precise identification of mineral element distributions within the oxide matrix. Overall, additional investigation of the random find patina might tell us a story about what happened with the coin after it left the mint, representing a secondary goal of the current research.

## 2. Materials and Methods

### 2.1. Sample Descriptions

**Figure 1:**
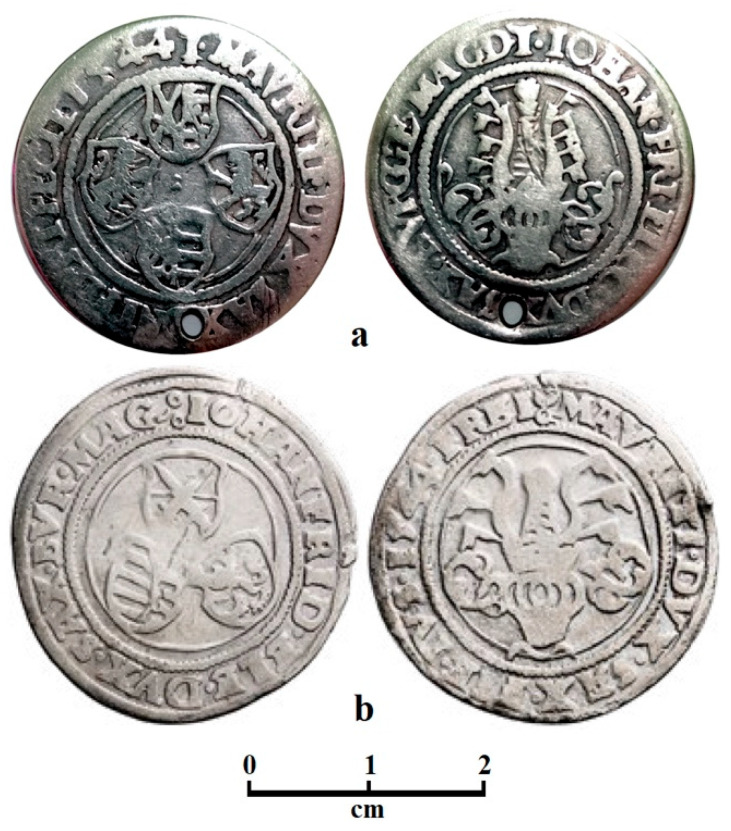
Photographs of the investigated Saxon quarter thalers issued in Saxony. John Frederick I and Maurice (1541–1547), 1544, ¼ thaler. (**a**) Mint: Buchholtz und Schnneeberg, Mintmaster: Sebastian Funcke (1534–1569), Mint mark: T. (**b**) Mint: Freiberg, Mintmaster: Hans Weller gen. Molsdorf (1541–1545), Mint mark: lime tree leaf.

The subject of the present research is a Saxon quarter thaler issued in 1544. The main sample is a randomly discovered piece from a Transylvanian flea market with heavily worn marks, revealing the silver alloy surrounded by black patina on the unworn areas, as shown in Figure 1a. It has a well-preserved condition, but the cleaning conditions are not known since it is a random find. It was subjected to a simple ultrasound cleaning for 15 min in bi-distilled water to remove any actual contaminants.

(**a**) **Obv.:** IOHANnes FRIdericus ELECtor DVX SAXoniae BVRGGravia E MAGDeburgensis T; Helmet of the Duchy of Saxony; crest above the helmet—a tuft of feathers protruding from the crown of the ruff; mintmark: letter T.

**Rev.:** MAVRITIus DVX SAXo-NIae FIERI FECIT 1544 T; four shields: electoral swords of the Arch-Marshall of the Reich and belts with the wreath of the Duchy of Saxony (1), climbing lion in the crown of the Landgraviate of Thuringia (2), Eagle with belts of the Burgravy of Magdeburg (3), and Margrave of Meissen (4). The legend reads: Maurice, Duke of Saxony, had minted [this coin] in 1544.

Silver; 29 mm; 6.65 g (because of the pierced hole and heavy wear); Keilitz-Kohl 208, random find.

Mintmaster; Sebastian Funcke, Mint: Buchholz (1529–1551), Schneeberg (1535–1569).

(**b**) **Obv.:** IOHANnes FRIDericus ELEctor DVX SAX BVRGgraviae MAGdeburgensis; three shields: electoral swords of the Arch-Marshall of the Reich (1), climbing lion in the crown of the Landgraviate of Thuringia (2), and belts with the rue wreath of the Duchy of Saxony (3).

**Rev.:** MAVRITIus DVX SAXoniae FIEri IVS 1544 FREI ♤; helmet of the Duchy of Saxony; crest above the helmet—a tuft of feathers protruding from the crown of the ruff; mint mark: lime tree leaf.

Silver; 29 mm; 6.81 g; Keilitz-Kohl 135, Slg. Merseburger 527; inv. no.: MG/N/675/711. Collection: Museum of Archaeology and History in Glogow (Poland). See the catalogue on page 112, no. 51 [34]. Mintmaster: Hans Weller (1541–1545), Mint: Freiberg (1541–1545).

Archeological reference is ensured by a Saxon quarter thaler issued in 1544 found in the Glogow Hoard, as shown in Figure 1b. It was found in a very well-preserved state and professionally cleaned under standard procedures of the museum (briefly: ultrasound cleaning in bi-distilled water to remove the remnant soil particles, followed by chemical cleaning with a solution of EDTA 15% to remove the oxide crust, followed by a final ultrasound cleaning in bi-distilled water for complete washing of the cleaning reagent). It is part of the permanent exposition of Glogow Museum of Archaeology and History [38].

### 2.2. Physicochemical Investigations

The elemental composition of the archeological reference was measured at the exhibit site by the X-ray fluorescence (XRF) method using aS1 TITAN 800 (Bruker, Karlsruhe, Germany). The elemental detection ranges from Mg to U, allowing precise investigation of narrow areas on the coin surface, ensured by an 8 mm collimator. XRF measurements were performed using the “Precious Metals” subroutine on two different sites on the obverse and reverse. Unfortunately, the archeological reference could not be subjected to further physicochemical investigation because it cannot be removed from the permanent exhibit in the museum.

The randomly discovered coin was subjected to a complex nondestructive physicochemical investigation as follows. XRD was effectuated with a Bragg–Brentano diffractometer produced by Bruker Company, Karlsruhe, Germany), model D8 Advance, with copper monochrome radiation Cu kα radiation (1.540562 Å). The diffraction speed was about 1 °/min in the 2θ range of 20–80°. The diffraction peaks were identified and analyzed with Match 1.0 software equipped with a PDF 2.0 database (Crystal impact Company, Bonn, Germany).

The patina components were examined through Mineralogical Optical Microscopy (MOM) under cross-polarized light with a Laboval 2 microscope produced by Zeiss Company (Oberkochen, Germany), equipped with the digital image capture system Sony 14MPX (Sony company, Minato, Japan).

SEM morphological investigation was carried out with a Hitachi SU8230 microscope (Hitachi Company, Tokyo, Japan) at an acceleration of 30 kV in high-vacuum mode. The elemental composition was assessed through the EDS method operated with the X-Mas 1160 EDX detection module (Oxford Instruments, Oxford, UK) installed on the SEM device.

Ultrastructural aspects were revealed by Atomic Force Microscopy (AFM) performed in tapping mode on a JSPM 4210 Scanning Probe Microscope produced by Jeol (Tokyo, Japan). The sample’s surface was tapped with an NSC15-Hard cantilever, with a resonant frequency of 325 kHz and a force constant of 40 N/m. The topographical and morphological details were analyzed with dedicated software WinSPM 2.0 (JEOL, Tokyo, Japan), ensuring the measurements of particle and crystallite sizes as well as surface roughness. At least three different sites on the sample surface were investigated to ensure scientific relevance.

## 3. Results

### 3.1. Silver Alloy and Patina Phases

**Figure 2:**
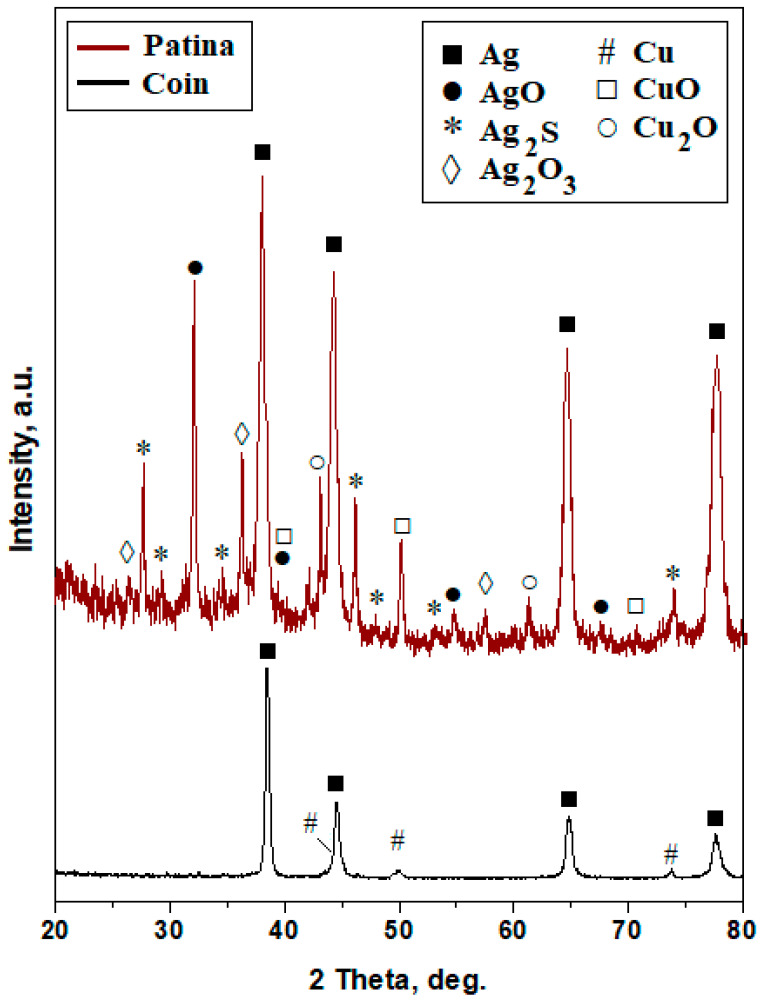
XRD patterns obtained for the pierced random find from Transylvania and its patina layer.

The first step of the XRD investigation was carried out on the median site of the coin surface, which was centered with the helmet (Figure 1b) directly on the incident X-ray to reveal the alloy phases. It results in very intense and narrow peaks for silver (e.g., α phase), indicating the dominance of silver grains, as shown in Figure 2. The copper peaks are very weak, indicating a very low content, and their slightly broadened aspect indicates that the copper amount (β phase) appears as thin lamellas within the eutectic grains. As a consequence, the randomly discovered coin has a pronounced hypoeutectic silver alloy. Applying the Relative Intensity Ratio (RIR) method [43,44] results in a roughly silver amount of about 92 wt.% and a copper amount of 8 wt.%. This content is considerably increased compared to the Joachimsthaler etalon [29] and requires further investigation for confirmation.

The other XRD diffraction site was positioned on the black areas beside the lateral inscription on the coin surface, just beside the inscription of the year 1544 in Figure 1a. The diffraction pattern in Figure 2 has very abundant peaks with different shapes and intensities merged into a complex situation. The pattern is dominated by silver peaks, which are detected partly beside the patina area and partly from beneath the oxide layer, which is not very thick. The silver peaks are very intense and narrow, while the oxides are less intense and slightly broadened, indicating the prevalence of small microstructural clusters, which are perhaps formed by nano-crystallites. The silver peaks were accepted from the RIR calculation of the patina pattern; the obtained amounts are presented in Table 1. The natural oxidation processes within the patina layer eliminate mechanical strain in the compounds’ crystalline lattice, allowing the crystallite size to be determined using the Scherrer formula [45,46].

**Figure 3:**
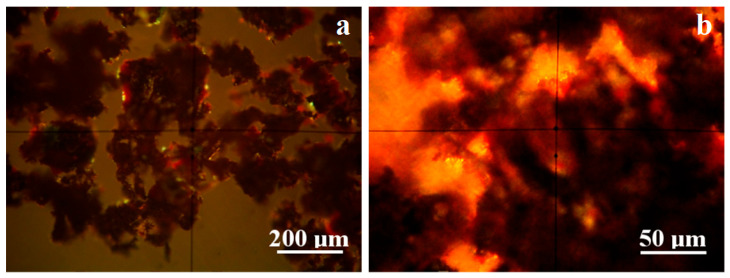
MOM images of the patina product: (**a**) overview and (**b**) microstructural detail.

A small quantity of patina was gently removed from the coin surface without altering the macroscopic aspect. It was spread on a glass slide and inspected with optical mineralogical microscopy under cross-polarized light. Each mineral has a specific color, allowing a satisfactory correlation with the cluster’s micro-size, as shown in Figure 3.

The patina is dominated by the presence of argentic oxide mixed densely with silver rust, generating black clusters with bright yellow spots, as shown in Figure 3a. Silver sulfide crystallized as argentite is hard to distinguish, requiring high magnification, as in Figure 3b, where it appears as very dark polyhedral clusters resembling monoclinic crystallization. Black tenorite clusters are tightly bonded to cuprite formation, with a brown-reddish shade, as clearly observed in Figure 3b.

Another significant observation from the cross-polarized light microscopy of the patina is the complete absence of soil mineral particles. A long resting of artifacts in the ground causes merging of mineral particles into crusts deposited over ceramic fragments [39], which are prone to being embedded into the oxides formed by the corrosion of metallic artifacts [40]. For instance, quartz would have appeared as boulder-like particles with green-gray nuances; clay particles, like kaolinite (white–blue) and muscovite (pink) [47], would have been detected. Their absence represents an important discovery regarding what happened to the coin after it was released from the mint.

Thus, the randomly discovered four-shielded Saxon quarter thaler looks like it was consistently oxidized in the atmosphere and in contact with human body parts (e.g., mostly hands, but the pierced hole most likely indicates that it was worn as a pendant, which was surely in contact with the skin’s surface for a long time). This explains the compactness and shiny aspects of the patina layer.

### 3.2. Morphology and Elemental Composition

**Figure 4:**
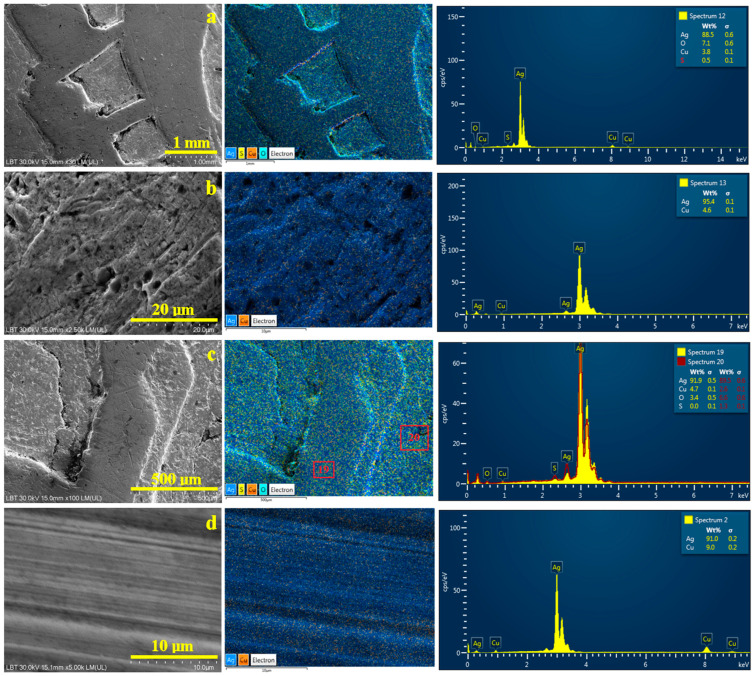
SEM-SEI images of the pierced random find from Transylvania: (**a**) general view, (**b**) worn alloy microstructure, (**c**) patina layer junction with the worn alloy surface, and (**d**) bulk alloy microstructure. BSE with the elemental map is taken in the same position as the SEI image and is displayed on its right side along with the EDS spectrum.

The morphological features of the coin’s surface are optimally evidenced by the Secondary Electron Images (SEIs) with a wide field depth, which ensures a proper focus at both low and high magnifications, as shown in Figure 4.

Backscattered Electron Images (BSE) taken at the same place as SEI images allow for a proper elemental investigation of the site, revealing an element distribution map superimposed on the morphological details. Specific colors are assigned to each element: blue to silver, orange to copper, yellow to sulfur, and turquoise to oxygen. BSE elemental maps are presented on the right side of each SEI image, along with an EDS spectrum for all image surfaces, with the elemental composition expressed in weight percent. EDS spectra can be acquired from specific regions within the BSE image, with each area clearly marked to indicate the corresponding spectrum number.

Figure 4a shows the overall surface of the randomly found coin, highlighting a macrostructural detail of the helmet ornaments. Their top edge is heavily worn, presenting a smooth, uniform surface. The downward sides and lower areas remain unaffected by wear, preserving the morphological details imprinted during the sticking process. The lower side of the downward slope has strong black marks of patina. The lower unworn surfaces of the coin display the roughness created by the die when the details were stamped onto the silver sheet. The elemental map has a uniform blue color because of the increased amount of silver, spotted with small orange dots corresponding to the copper distribution within the eutectic grains. The dark regions of the patina layer seen in the SEI image now appear turquoise, reflecting a higher oxygen content. The overall elemental composition of the investigated site in Figure 4a has 88.5 wt.% Ag, 7.1 wt.% O, 3.8 wt.% Cu and 0.5 wt.% S. This confirms the XRD findings on the composition of both the alloy and the patina layer. All expected elements were present in suitable amounts, with no additional contaminants detected.

The oxidized regions need to be distinguished from the fully clean alloy surface to accurately investigate the alloy’s characteristics. Therefore, optimal sites were identified on the worn area at a magnification of 2500× to reveal the grain microstructure and element distributions, as shown in Figure 4b. The microstructure is dominated by the presence of α phase grains with elongated shapes oriented in the rolling direction. Their lengths range from about 10 to 15 µm, and their widths are situated around about 5–8 µm. These are intercalated with relatively tiny grains with an eroded aspect belonging to eutectic formations. It is relatively difficult to establish their relative size because of corrosion pits associated with material loss; however, they fall within the size range of α phase grains. The elemental map is uniformly colored in the specific color of silver without any oxidation marks, while the small orange spots appear on the eutectic grains surrounding the corrosion pits. The resulting elemental composition is 95.4 wt.% Ag and 4.6 wt.% Cu, surpassing the standard set by the Joachimsthaler benchmark [29]. Saxony is well known for its silver mines developed since the Middle Ages [48]; would they really be so lavish as to issue coins with such a high silver content, or could something else have occurred? The silver content of 92 wt.% calculated from the XRD pattern is slightly lower than the EDS value detected from the surface of the coin. However, XRD radiation has a greater penetration depth than the accelerated electron beam of the SEM device and may collect information from deeper layers of the alloy.

The literature data indicate that corrosion processes affect the elemental composition of bronze artifacts, typically showing an apparent increase in tin content due to copper loss from oxidation [49], which may occur in silver alloys exposed to similar conditions. Ioanid and collaborators reveal that the oxidized areas on medieval coins present an appreciation of copper levels [50]. Subsequently, the removal of copper via corrosion products results in an apparent increase in silver content in the underlying clean substrate. Beck et al. confirm that silver content appreciation within an artifact’s surface as a consequence of copper removal through the cleaning process may affect XRF and other investigation techniques that do not receive the signal from deeper layers [51].

Perfectly cleaned worn silver surfaces of the coin might be scarcer in copper content, which might increase in the less cleaned areas. Therefore, Figure 4c shows the microstructural detail of an area gradually affected by corrosion, which trespasses in a mild manner onto the worn area and with a consistent patina layer deposit on the downward slope base. The EDS spectrum 19 in Figure 4c clearly reveals an increase in the Cu content up to 4.7 wt.%, and the silver amount decreases accordingly. Mild oxidation of these features is proved by oxygen levels of 3.4 wt.%. A dark, thick patina deposit tested by spectrum 20 reveals a strong increase in oxygen levels to 6.6 wt.% and detects significant traces of 1.3 wt.% S. As a result, we must acknowledge that the unknown cleaning process of the randomly found coin has increased the silver content in its outermost layer.

The composition of the bulk must be established, and minor invasive treatment was necessary. The pierced rim was gently polished by removing a few micrometers with a jeweler’s chisel. The overall aspect of the coin was not affected, but the elemental analysis of the bulk was done in good conditions, as shown in Figure 4d. The SEI image reveals the parallel scratches induced by the chisel, making it difficult to observe the microstructural aspect of the grains, but the elemental map reveals a densification of fine orange spots where the eutectic grains appear. Thus, the copper amount increases up to 9.0 wt.%, and the silver amount is 91.0 wt.%. The bulk alloy seems to have a better title but is still closer to the Joachimsthaler etalon, a fact confirmed by the XRD measurements. On the other hand, the patina and the oxidation processes that lead to its formation require detailed investigation in a dedicated sub-study.

**Figure 5:**
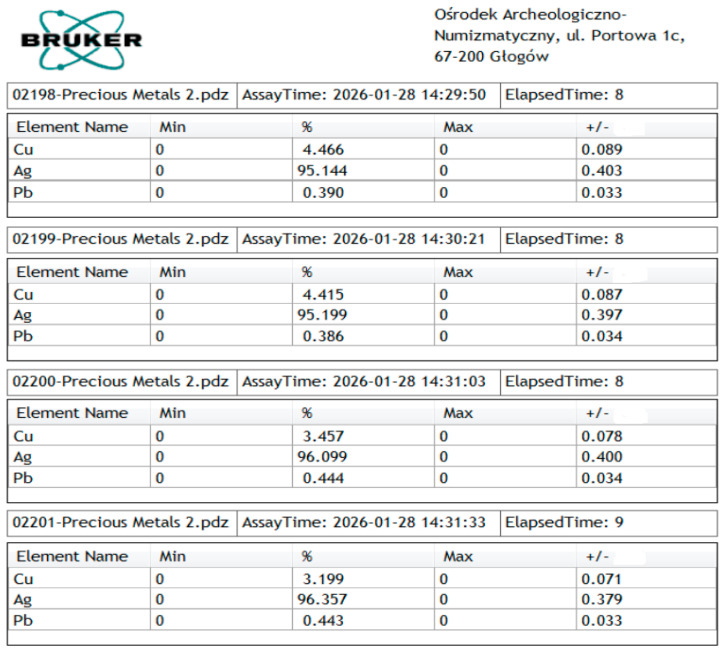
Snapshot centralization of XRF measurements effectuated on the Saxon quarter thalers issued in 1544 found in the Głogów Hoard: measurements no. 02198 and no. 02199 are taken on the reverse, while measurements no. 02200 and nr. 02201 are taken on the obverse (original XRF data files have extension “.pdz”).

The archeological reference from the Glogow Hoard was investigated in detail with XRF, obtaining pertinent values on the obverse and reverse. The experimental data snapshots are shown in Figure 5.

The coin reference discovered at the archeological site was cleaned according to museum standards, with care for preserving the optimal state of the surface. Thus, the silver amount is situated between 96.09 and 96.35 wt.% on the obverse and 95.14 to 95.19 for the reverse. The copper amount varies accordingly, with more on the reverse than on the obverse, as shown in Figure 5. This observation aligns well with the overall appearance of the coin’s reverse, which still retains minor traces of patina, unlike the obverse, which is clean and free of stains. Consequently, the XRF measurements confirm the dependence of the copper amount in the coin’s surface on the cleaning process, but the influence is less effective than that exerted by the unknown cleaning process that happened to the randomly discovered one. The resulting mean content of silver in the archeological etalon was about 95.69 wt.%. Corroborating data on both coins suggests that the quarter thaler with four shields has a slightly lower silver amount of only 95.40 wt.%, indicating a difference of 0.29 wt.%, which could still generate profit for its issuer in 1544.

The bigger differences consist of significant traces of lead measured in the archeological etalon, which ranges from 0.38 to 0.44 wt.%, with an average content of 0.41 wt.%. This is not enough to alter the characteristics of the silver alloy and overall aspect of the coin, but Pb is surely segregated at the grain limits between the α phase and eutectic. Unfortunately, this sample cannot be investigated by SEM-EDS at the current phase of the investigation, but it may be considered in the future.

Since the randomly discovered coin is lead-free, its silver alloy might be elaborated at a different place (mint) or at the same place but within another charge with ore originating from another source than the archeological etalon. Briefly, this observation suggests that the investigated coins might have been struck in different mints from Saxony, one most likely controlled by Duke Maurice and the other one controlled by Duke Elector John Frederick I. The material characteristics identified in the current research must be correlated with historical data from the literature regarding the silver ore source and supply chain of the mints controlled by both cousins facing these coins. Lead often appears in alloys and silver coins because it was originally present in ores, remained after refining processes, came from recycling, or originated from impurities during the addition of additives. Pure silver remains after removing lead oxidized to oxides, a cupellation process [52,53]. Thus, minor traces of lead may result from the cupellation process and should not be interpreted as evidence of intentional addition. These possibilities can only be confirmed through the examination of a larger number of coins from this period.

### 3.3. Patina and Corrosion Aspects

**Figure 6:**
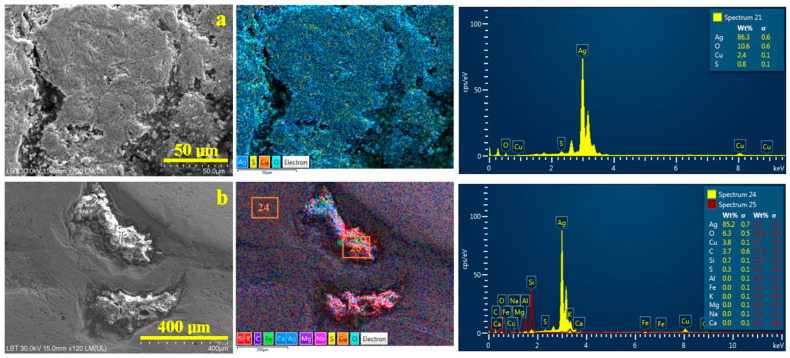
SEM-SEI images of the patina layer of the pierced random find from Transylvania: (**a**) microstructural detail on the oxidation layers and (**b**) mineral dust particles embedded in the patina layer. BSE with the elemental map is taken in the same position as the SEI image and is displayed on its right side along with the EDS spectrum.

The detailed investigation of the patina layer observed on the randomly discovered Saxon quarter thaler is less representative for the determination of silver title, but it reveals precious clues about what happened with this coin after it was pierced. SEM microstructural details with their additional elemental information are displayed in Figure 6.

The central area of Figure 6a reveals a group of a few α phase grains with polyhedral–elongated shapes because of the rolling process, with their borders well connected to each other. Elongated eutectic grain shape is more visible because of their advanced corrosion etching, revealing a mean length of about 15 µm and a width of about 10 µm. The elemental map reveals a uniform distribution of oxygen, proving that all observed features are deeply oxidized, a fact substantiated by the large amount of 10.6 wt.% O. The sulfur content is significantly high at 0.8 wt.%, in good agreement with the XRD observation.

The patination process acts in a relatively uniform manner over the entire coin surface through slow oxidative processes that affect the microstructural features. Hence, silver has a more noble position than copper on the reactivity scale; the eutectic grains are affected by the corrosive process, and afterward, pure silver α grains are affected. The literature reveals that the most corrosive agents are water and hydrogen sulfide, both in the atmosphere and underground.

For instance, Tran et al. reveal that the underground atmosphere containing hydrogen sulfide around a copper specimen with a humidity ranging from 35 to 75% first facilitates copper oxide formation, followed by alteration to copper sulfide [54]. The open-air exposure of copper-based alloys is affected by humidity and rainwater, as well as sulfur-based compounds like gaseous hydrogen sulfide and aqueous solutions of H_2_SO_4_ induced by acid rain mechanisms involving SO_2_ emissions cumulated in higher layers of the atmosphere [55].

Thus, open-air corrosion depends on local environmental aspects and pollutant type. However, the literature mentions the following types of compounds in the open-air patina of copper: oxides, e.g., cuprite, tenorite and spertiniite Cu(OH_2_), copper sulfide, sulfates, chlorides and nitrogen compounds like nitrates and nitrites [56]. This fact is strongly related to acid rain. Moreover, it was shown that the oxidation process is the first step of open-air patination, involving rounded oxide clusters ranging from submicron size to about 1 µm, with a coalescence tendency to form a compact layer after longer exposure; sulfates and other salts formed as a consequence of acid rain form dendritic deposits crystallized over the dense layer of copper oxides [57]. Recent studies reveal that the copper oxidation process occurs at the nanoscale, involving oxide nano-crystallites developed on the polished copper surface [58]. This fact is in good agreement with the crystallite sizes obtained through the Scherrer formula on XRD patterns.

The silver oxidation process also depends on the exposure environment, but the less reactive state of silver protects it against direct oxidation through water or high humidity exposure. Thus, its patination process occurs through the reaction with hydrogen sulfide, which forms a compact layer of silver sulfide Ag_2_S that further interacts with oxygen and water to form silver oxides [59]. Refined silver microstructures and nanostructures like silver nanoparticle deposits are prone to form nanostructural layers of Ag_2_S with rounded–elongated shapes, as observed in thin cross-sections obtained by TEM [60]. SEM investigation of such a silver sulfidation process reveals the development of small micro-clusters formed by fine nanoparticles, which progressively grow with the hydrogen sulfide concentration [61]. Their relative elongated shapes relate to monocline crystallization. Another typical habitus for Ag_2_S is represented by cubic-derived shapes like rectangular or octahedral particles [62].

The worn surface of the pierced coin is largely free of oxides, while the lateral areas near the patina deposits exhibit a thin oxide layer, visible as a subtle turquoise hue in the elemental map shown in Figure 4c. It corresponds to the thinner silver oxide layer mentioned in the literature that occurs on rich silver alloy surfaces accompanying copper oxides [59,60]. Deeper into the patina layer, the eutectic grains are severely fragmented by the advanced corrosion of the copper lamellas, subsequently disorganizing and corroding the silver lamellas and generating a broken structure like the one observed in the right lower corner of Figure 6a. Pure silver α grains are more resistant to the destructive action of corrosive agents because of their compactness and lower reactivity. The central site of Figure 6a shows such α grains covered with a thin corrosion product based mainly on Ag_2_S, evidencing a microstructure of the corrosion grains similar to the one in the literature [61]. Oxygen presence in an elemental map of this area suggests that silver sulfide was progressively altered to silver oxides. The corrosive destruction of eutectic grains and partial alteration of α grains require high magnification associated with topographic sensing of the affected surface, which should be accomplished through AFM investigation.

**Figure 7:**
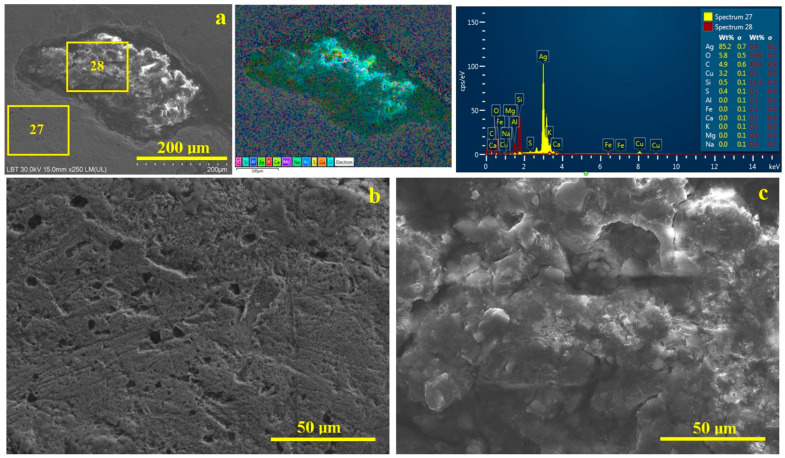
SEM images of microstructural detail of mineral inclusion within patina: (**a**) mineral particle embedding, (**b**) patina microstructure, and (**c**) mineral microstructure. BSE with the elemental map is taken in the same position as the SEI image and is displayed on its right side along with the EDS spectrum.

Before viewing the ultrastructural features regarding the pierced coin’s corrosion, it is worth noting that a few random mineral inclusions in the patina layer were detected, especially in close proximity to the hole, as shown in Figure 6b. These mineral inclusions appear well attached to the patina layer with a superficial embedding. They are formed by a dense mixture of boulder-like particles mixed with fine lamellar ensembles. The elemental analysis clearly indicated that these mineral inclusions are based on O 42.4 wt.%, Si 10.7 wt.% and Al 4.0 wt%, clearly indicating the presence of quartz and phyllosilicates like kaolinite and explaining the presence of K, muscovite, and moderate traces of Fe, Mg and Na. A significant amount of carbon, 37.9 wt.%, is distributed firstly to 0.3 wt.% of Ca, representing calcite fractions, and the other amount most likely relates to organic matter associated with the mineral inclusion, which is really strange. A detailed view of the mineral inclusions detected in our coin patina is presented in Figure 7.

The outer patina layer has the same microstructure and elemental composition as the other investigated areas on the coin surface, with a moderate amount of S of about 0.3 wt.%, in good agreement with previous measured levels. It is very interesting that the sulfur content significantly decreases to 0.1 wt.% in the mineral inclusion area, and carbon excess occurs. The literature data reveal a uniform distribution of mineral elements within the patina surface of metallic artifacts affected by corrosion in their presence, for example, Ca atoms related to mineral content had a uniform distribution on the corroded area of a silver Samarian coin, but unfortunately, the carbon distribution was not displayed to determine its possible correlation with calcite [63].

The patina layer has a smooth and uniform surface, with wear scratches very similar to the worn pattern observed on the silver surface, as shown in Figure 7b. The dense corrosion matter has small microstructural pores ranging from only a few micrometers to about 15 µm in diameter. The microstructure of the mineral inclusions is more clearly visible in the detail shown in Figure 7c, revealing boulder-like particles with multiple sharp edges that are rich in Si and O, as indicated by the elemental map.

They correspond to a literature observation revealing the same morphological features of quartz [47]. The upper median side of Figure 7c reveals a rounded boulder-like particle, corresponding to a significant accumulation of C and Ca and representing a calcite particle. Both quartz and calcite particles are tightly surrounded by a dense mass of fine lamellar particles belonging to kaolinite and muscovite, mineral binders typically found in street dusts [47] and clayey soils [44]. Thus, the limited distribution of minerals within the coin’s patina suggests they may have become embedded while the coin was in circulation. This hypothesis is substantiated by mineral inclusions positioned close to the pierced hole, where the silver surface wear was protected by the local rim on the obverse and the depression on the reverse, keeping the patina layer thicker than in other areas of the coin.

**Figure 8:**
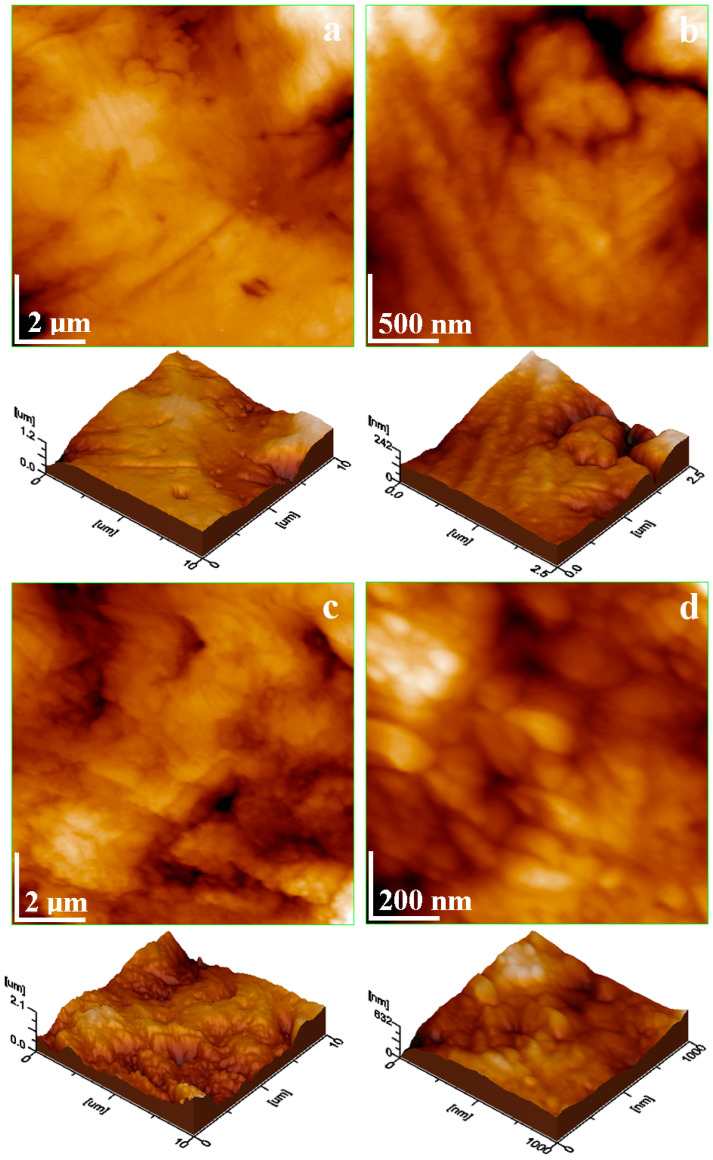
AFM images taken of the worn alloy surface: (**a**) fine microstructural detail, (**b**) nanostructure, and the patina surface, (**c**) fine microstructural detail, and (**d**) nanostructure. Three-dimensional profiles are presented below each topographic image.

AFM ultrastructural data reveal α grains in the central site of Figure 8a, evidencing random scratches caused by the coin’s circulation. Its consistency is dense and uniform, proving α phase cohesion and ensuring a roughness of only 108 nm. An eutectic grain border occurs on the upper right side of the topographic image in Figure 8a, revealing a moderate topographical depression. An eutectic lamellar structure can be observed by a succession of thin parallel lines, with the upper right corner indicating a width of approximately 500 nm. The nanostructural detail in Figure 8b evidences a dark depression with a descending crack to the right, indicating corrosion product loss within the corroded copper lamella, while a silver lamella fragment is still attached to the α grain border. This material proof substantiates the coin’s patination mode and, subsequently, the silver amount enriched in the cleaned surfaces.

**Figure 9:**
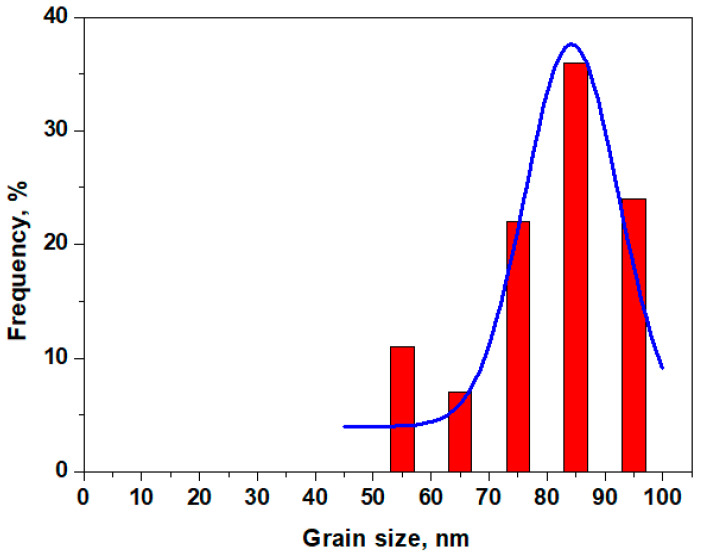
Nanoparticle distribution within the patina layer. The blue line represents the Gaussian fit of the distribution trend.

The corrosion product topography has certain asperities, as shown in Figure 8c, with small pores ranging from about 0.8 to 1.2 µm, in good agreement with the smallest pores detected by SEM in Figure 7b. The tridimensional profile reveals surface waviness, which leads to a roughness of about 240 nm, considerably increased compared to the silver areas. The nanostructural detail in Figure 8d clearly suggests that the patina layer is made of corrosion product nanoparticles embedding each other in a dense matrix, confirming the crystallite sizes calculated from the XRD patterns. The nanoparticle distribution, as obtained by Image J software version 1.53k, is presented in Figure 9.

The dominant diameter is 85 nm, corresponding to CuO crystallites, followed by a diameter of 95 nm, corresponding to AgO crystallites. A significant frequency occurs for a diameter of 55 nm, which corresponds to Ag_2_S. Overall, the nanoparticle distribution confirms the XRD crystallite sizes and the detected compound amounts.

The silver sulfide and silver oxide nanostructural distribution indicates a progressive germination, possibly facilitated by the bearer’s sweat, which contains minor traces of hydrogen sulfide and other sulfur compounds related to dietary habits like consumption of onion, garlic and eggs, correlated with skin enzymatic activity [64,65]. Such information suggests that the pierced coin was most likely worn as a pendant or knitted into hair, like in nomadic population habits [66].

The archeological reference coin (e.g., three-shielded quarter thaler) belongs to the Glogow Hoard, which was treasured in textile bags deposited in wooden chases. They were buried under basement floors, most likely after 1656 (the latest year bared by a coin from the hoard), and discovered during archaeological excavations in 2004. Hoard’s photograph, taken just after its discovery, reveals a well-preserved silver surface with a consistent green patina [38]. Green corrosion products relate to eutectic grains, which react with carbonate-rich environments, generating malachite, in good agreement with the literature data [6,67]. The general aspect of resting soil indicates a high amount of calcium carbonate as a construction material that progressively reacts with water infiltration, facilitating the carbonation of copper lamellas within eutectic grains. Thus, as observed in Photograph 7 in the exhibition catalogue [38], the green deposit is very thin, less cohesive, and easily removed by a moderate cleaning process in the museum’s restoration room, preserving the alloy bulk composition without significant enrichment of the pure silver α phase. The three-shielded quarter thaler obverse reveals two darkened patina spots on the right side close to the border, as shown in Figure 1b, suggesting the mild cleaning of the coin. The patina comparison indicates a longer circulation of the pierced coin, while the hoarded one was kept in a wealthy man’s vault, thus having fewer circulation marks.

## 4. Discussion and Numismatic Significance

During the division, the rules of Saxony–Elector and Duke were agreed upon for the joint extraction of silver and minting of coins by both dynastic lines. The discovery of silver deposits near Schreckenberg led to the founding of mining towns, such as Schneeberg (1473), Annaberg (1496), Buchholz (1501), Scheibenberg (1522), and Oberweisenthal (1527). New mints were established in Schreckenberg, Schneeberg, Annaberg and Buchholz, which played an important role in the development of the Saxon monetary system [33,34]. In these mints, new silver denominations were introduced, including Mutgroschen, Zinsgroschen or Schneeberger, valued at 1/21 of the gold coin, Rhenish Gulden. According to the minting ordinance of 1498, these coins were struck at a rate of 88 pieces from the Erfurt mark (233.81 g) of silver of 7⅔ lot fineness (479/1000). The ordinance also introduced the so-called Schreckenberg groschen, with a value of 3 Zinsgroschen. The Schreckenberg groschen corresponded to 1/7 of a Rhenish Gulden and contained approximately 3.92 g of pure silver (about 86.1%).

However, the star was a new coin minted in silver gulden, equivalent to the gold Rhine Gulden; therefore, at the very beginning, it was also called gulden, which is the same as the unit of gold. Saxony thus gained a coin that could compete with the gold denominations (Rhenish Gulden or florin). In 1500, Elector Frederick III the Wise and his brothers signed the Leipzig Monetary Ordinance to introduce this new high silver denomination into official monetary circulation and to prevent its export abroad. The exchange rate of the silver gulden (Guldengroschen) was maintained at 21 Zinsgroschen and 3 Schreckenberg groschen. Foreign coins were forbidden in circulation except for Prague groschen. Even payment in Rhenish Guldens was largely impeded. Since then, only the following silver denominations have been officially in use in Saxony: Guldengroschen, ½ Guldengroschen, Schreckenberger (=1/7 Guldengroschen), Groschen (=1/21 Guldengroschen), ½ Groschen, Pfennige (=1/12 Groschen), and Heller (=1/2 Pfennige). Guldengroschen contained 93.77% and 27.41 g of pure silver (the gross weight: 29.23 g). Therefore, the ratio of gold to silver at that time can be determined as 1:10.8. The production of silver guldens increased from 1507, and from the turn of the first and second decades of the 16th century, they became the subject of long-distance trade. It should also be remembered that the increase in the production of silver gulden was determined not only by new discoveries of silver deposits but, above all, by using machines for cutting blanks and striking coins. The ordinance from 1500 was an agreement between all three representatives of the Wettin dynasty ruling at the time, which is why the first silver gulden coins depict three men: on the obverse, Frederick III the Wise (the main representative of the Ernestine line, who also held the title of Elector of the Reich), and on the reverse, Frederick’s younger brother John and, opposite him, their uncle from the Albertine line, Albrecht, and after Albrecht’s death, Prince George. This rule was followed in Saxon silver guldens for several decades. Although the first ones do not bear dates, their production can be traced using the marks of the mint masters in Annaberg, Buchholz, Freiberg, Leipzig, Schneeberg and Zwickau until 1525. It was only later electors who marked their silver guldens with dates [35].

Saxony’s position was so strong that in 1522, Emperor Charles V (1516–1565) issued the Nuremberg Edict, which included silver guldens minted to the Saxon standard and their fractions. Exactly eight guldens were to be minted from one “raw mark” of silver, and the groschen was 1/21 of a gulden. The minting ordinance issued two years later in Esslingen maintained the Saxon thaler standard. The gulden eventually became the thaler, an important currency of the Holy Roman Empire, but linked to other currencies, such as the Czech and Silesian large coins [35].

After 1525, a dispute broke out between the two Saxon lines. The cause was the reduction in the fineness of the Rhenish Gulden and the associated increase in the price of gold, as well as the export of coins with high precious metal content. Between 1530 and 1533, minting cooperation between the two Saxon lines was suspended because the Protestant Elector John I (1486/1525–1532) and the Catholic Duke George the Bearded, formerly known as George the Rich (1500–1539), could not reach an agreement. The Elector demanded a reduction in the silver content of the coins, while the Duke insisted on maintaining their existing value. As early as 1530, it became apparent that the Rhenish Gulden, whose silver equivalent was the silver gulden, was losing much of its value. At a conference of the estates in Augsburg in 1533, it was stated that “the good old coins were broken, sawed, sold by the cartload, and replaced with worse and worse coins”.

The Elector, who closed the joint mint in Schneeberg and temporarily opened a mint in Zwickau in 1530, ordered—without official announcement—that coins be minted there according to a lighter standard, while Prince George maintained production in Freiberg, Leipzig and Annaberg according to the old, heavier standard, which by 1533, led to the parallel circulation of coins of varying quality. Prince George argued that the legality of circulation required maintaining the value of the silver gulden coins used by the population. Elector John, on the other hand, claimed that high-quality Saxon silver guldens were detrimental to society because they were taken out of the country by users and replaced with money of lower value [32]. The conflict between the Protestant Elector and the Catholic Duke also had religious and political roots. In 1531, several Protestant princes, led by the Elector of Saxony (John, 1525–1532, and later John Frederick, 1532–1547) and Philip I, Landgrave of Hesse (1509–1567), joined forces in the Schmalkaldic League against the religious policies of Emperor Charles V, who demanded the former catholic church property for himself [37].

Finally, on 17 July 1531, the estates of both parts of Saxony (eastern Meissen, which belonged to the Albertine line) and western Thuringia (belonging to the Ernestine line) gathered in Grimm, where they passed the so-called Grimm Decree, demanding the standardization of the monetary system. Both the Duke and Elector agreed to the demands of their estates [32]. Unfortunately, the Elector John died in 1532, and negotiations on future coinage were interrupted. After his death, John Frederick I became Elector of Saxony. For the first ten years, John Frederick shared the rule (but not the Electoral dignity) with John Ernest, his younger half-brother. In 1542, John Frederick I decided to rule alone and ceded the Franconian areas of the Wettin family lands (Coburg and Eisfeld) to John Ernest. As it was later described, this had an impact on monetary emissions.

On 18 November 1533, the estates decided to resume joint coinage and issued an explanation to the Grimm Decree, which resulted in the resumption of coin minting for the new Elector John Frederick, his co-ruler John Ernstand, Duke of Saxony, and George the Bearded, from 1534. A reduction in the minting rate of ½ ort per mark was agreed upon, reaching a compromise: silver guldens retained their previous silver content, while smaller denominations were devalued in line with the decline in the value of the Rhenish Gulden, causing the loss of their equivalence. The ordinance of 20 January 1534, announced jointly by John Frederick and George the Bearded, together with the valuation of foreign coins, confirmed the new system with the name “Guldengroschen” given to silver gulden, raising its value to 22 Groschen. As a further consequence, the thaler system (24 Groschen) was established, as shown in Table 2, and some foreign coins that did not comply with the Saxon standard were withdrawn from circulation [32].

These arrangements remained in force until 1549. The Duke of Saxony from the Albertine line, George the Bearded, died in 1539. George’s sons had died earlier, without children. Therefore, after George’s death, the duchy passed into the hands of his brother Henry (1539–1541), although George desperately wanted to avoid this because Henry was a Lutheran. At that time, the duchy converted to Protestantism. Henry was succeeded in 1541 by his son Maurice (1541–1547).

The coinage of the Saxon Elector John Frederick and Prince Maurice (1541–1547) was based on the Rhenish Gulden and the Tyrolean–Saxon Guldengroschen. Both the Ernestine and Albertine lines were entitled to half of the mining revenues and to one side of the jointly issued coins. When Maurice of the Albertine line became duke in 1541, both rulers remained formally bound by the mint regulations of 1534; however, political tensions limited the frequency of joint coinage. Maurice struck coins together with Elector John Frederick at Annaberg, Freiberg, Buchholz, and Schneeberg. John Frederick demanded that Duke Henry and, from 1541 onward, his successor Maurice consent to placing the names and portraits of the Saxon co-rulers—John Frederick, John Ernest, and, initially, Henry, then later Maurice—on the coinage. Neither Henry nor Maurice agreed, maintaining that just as they were entitled to half of the mining profits, they were likewise entitled to one full side of the coin. Nevertheless, in 1541–1542, John Frederick issued coins bearing three names (¼ and ½ Guldengroschen) and, in the case of the Guldengroschen, three portraits at the Buchholz mint. No comparable issues are known from the ducal mints at Freiberg and Annaberg. The dispute was resolved when the Elector granted his brother John Ernest the administration of Coburg, after which he withdrew from the joint coinage arrangement. In a letter dated 1542, the Elector noted that he had settled matters with his brother, who would no longer be involved in minting; accordingly, his portrait was removed from the coins [37].

In 1542, both Saxon governments (Electoral and Ducal) decided to set the value of the Guldegroschen at 25 groschen and to completely cease the minting of their own groschen and Dreier coins, because the small coin was overstated in intrinsic value, and was therefore exchanged for smaller coins, removed from the country, and taken to other mints, where it was melted down. All small foreign coins were banned, and only coins of higher denominations could be in circulation if they were of the same standard as Saxon coins. But this measure did not work. On 9 June 1542, the Mügeln Coinage Treaty (essentially published in the coinage regulations issued by Duke Moritz on 1 July 1542) was therefore concluded with the participation of Landgrave Philip of Hesse. The Guldengroschen was fixed at 24 Groschen. The shortage of small change was to be remedied by the reintroduction of the Zinsgroschen and Dreier coins. Contrary to the provisions of this treaty, Elector John Frederick unofficially reduced the fineness of the Guldengroschens minted in his mints in Buchholz and Schneeberg by two grains (approximately 7‰) until 1546. His Guldengroschens (thalers) no longer had the prescribed 903/1000 and were only 896/1000 fine. In this way, he hoped to personally benefit from the exchange of profits. However, Duke Maurice and the Estates rejected any deterioration in the coinage standard. Therefore, in order to distinguish the coarse coin types minted in his mints in Freiberg and Annaberg from those of the mints in Schneeberg and Buchholz, located in the Elector’s territory, in 1542, the Duke instructed the mint masters to mark whole, half, and quarter Guldengroschens not only with their mint marks but also with the initial letters of the mint names—ANB, FRI, FRIB, FREI, or FREIB (Figure 1b). In those days, when all mints, both electoral and ducal, minted coins bearing the names and images of both rulers, that was the only way to distinguish Duke Maurice’s good coin from the inferior coin of the Elector, thus sparing the Duke the accusations of debasing the coinage and justifying him by visual inspection [32].

The quarter thaler we are interested in was minted in Buchholz or Schneeberg, which were managed by Electoral mint master Sebastian Funcke (Figure 1a). The Bucholtz mint was established in 1505 during the reign of Elector Frederick III. It was located in the mining town of Bucholtz in the Ore Mountains (Erzgebirge) on the territory of the Ernestine line. However, the mining areas in the Ore Mountains remained under the joint administration of both lines. Annaberg and Buchholz were in this common belt. The profits from silver mining and coin minting were divided equally. The Buchholz mint was established for reasons of prestige; it produced only 10% of what the Annaberg mint produced and was destroyed by fire in 1544. The Buchholz mint was closed in 1547 and merged with the Annaberg mint [68]. The most important distinguishing feature on coins of this period from the Buchholz and Schneeberg mints is the letter T (often placed on the rim after the date). It refers to the master mint master Sebastian Funcke (1529–1551). The mint mark T was used in Buchholz until 1551 and in Schneeberg from 1535 to 1569.

Coins minted during the joint reign of John Frederick and Maurice, like the previous joint issues of the Electors and Dukes of Saxony, are a symbol of the so-called “hereditary community”, while the coats of arms and legends emphasized the hierarchy: John Frederick, as Elector, occupied the obverse and Maurice, as Duke, occupied the reverse. The management of the mint in 1544 was based on the joint mint ordinance of John Frederick I and Maurice, signed in July 1542. Although the mints were located in both territories, joint minting was established. However, John Frederick managed the mints in Schneeberg and Buchholz, while Maurice managed the mints in Annaberg and Freiberg. Quarter thalers minted in Freiberg and Annaberg feature three coats of arms, denoting the three oldest possessions: the Electorate of Saxony, the Duchy of Saxony, and the Landgraviate of Thuringia. The reverse of the coins minted in Buchholz and Schneeberg features a fourth shield, denoting the Bourgraviate of Magdeburg, which Elector John Frederick purchased in 1538 from the Archbishopric of Magdeburg for a considerable sum. The income from coinage fees was divided equally, which forced both parties to cooperate closely, despite their growing political animosity.

This fact is substantiated by the SEM-EDX and XRF measurements of the alloy composition of the cleaned surface of both coins, indicating a silver amount of about 96 wt.%. This is significantly higher than the standard value presented in Table 2, making us believe that the abnormal increase in the silver level on the coin’s surface occurs due to corrosion product removal through cleaning, which partly enriches the outermost microstructure’s layers in α phase grains. John Frederick’s pierced coin, as shown in Figure 1a, allows testing of the deeper layer without damaging the general aspect of the artefact, revealing a silver amount of 91 wt.%, in good agreement with the standard title in Table 2. Unfortunately, the coin exhibited in the Glogow museum cannot be tested in depth, but a similar composition may be expected.

Lead contained in the coin issued by Maurice indicates a different smelting process carried out by Hans Weller in the Freiberg mint. The lead amount of about 0.4 wt.% indicates 4 kg of lead introduced per ton of silver alloy. The small amount of lead found in the 1544 Freiberg quarter thaler from the Glogow Hoard is best understood in terms of historic silver refining practices rather than deliberate alloying in the mint. In the early modern period, especially in mining districts such as the Saxon Ore Mountains, most silver was obtained from argentiferous lead ores containing galena (lead sulfide), with lead and silver closely associated in the raw material. After smelting, the resulting lead-rich alloy was refined by cupellation, a process in which impure lead was heated in a furnace and oxidized to litharge (PbO), leaving behind purified silver; this method was widely employed to extract and purify silver from lead-rich ores in Europe before more modern techniques developed [68,69]. Although cupellation was designed to remove as much lead as possible, trace amounts often remained in refined silver because complete separation is metallurgically difficult; archaeological and metallurgical studies indicate that silver recovered by cupellation typically contains measurable lead remnants because of the refining process. These residual lead traces, detectable in microanalysis of historic silver alloys, therefore do not reflect intentional alloying in the mint but rather technological residue from the upstream refining of silver used as mint metal [48,49]. However, it can be assumed that the silver content in the quarter thaler with three shields is higher, as indicated in the literature on the subject, a fact substantiated by the difference observed in the XRF results on the archaeological etalon and SEM-EDX results obtained on the random finds. The high purity of the Ag–Cu alloy within Maurice’s coin confirms his rigorous attitude regarding monetary circulation, sustaining his ambition to increase his power and title.

Both quarter thalers left their mints in Buchholz or Schneeberg and, respectively, Freiberg in 1544, spreading in circulation [70,71]. Just after their emission, tumultuous times affected the circulation of people and coins. During the Schmalkaldic War (1546–1547), Prince Maurice of Saxony defected from the emperor, which led to the defeat and dissolution of the Schmalkaldic League. After the decisive battle of Mühlberg (24 April 1547), Elector John Frederick was taken prisoner and, because of the capitulation of Wittenberg, lost his electoral dignity and a significant part of his lands to Maurice. Thus, the electorate passed to the Albertine line, which also entailed changes in the organization and authority over the Saxon mints. Under this treaty, the Ernestine branch of the Wettin dynasty lost all its rights to the Saxon silver mines (except for the tithe from Schneeberg) and all mints. All mints operating up to that point became the exclusive property of Maurice, which ended the monetary union between the Ernestine and Albertine lines. From then on, he minted coins exclusively under his own name at the Annaberg, Freiberg and Schneeberg mints. The Buchholz mint was merged with the Annaberg mint, ending operations in Buchholz. Maurice also took over the town of Zwickau, which had previously belonged to the Albertine line [37].

The Saxon states encouraged Maurice to oppose the imperial monetary decree of Esslingen of 1524 and supported his efforts to introduce his own. On 27 March 1549, in Torgau, the elector issued his own monetary regulations, establishing the Erfurt mark as the basis of weight (like the medieval Cologne mark—233,856 g, but about 1 g lighter). Among other things, it was decided that:

1 Güldengroschen = 21 Groschen = 252 Pfennige;
1 Orts/Viertelgroschen = ¼ Gulden = 63 Pfennige;
1 Zinsgroschen = 12 Pfennige.

The reform also covered the weight parameters and fineness of individual denominations. Maurice reinstated the minting of halers and reduced the fineness of the Zinsgroschen, Dreier and Pfennig coins. The final division of coins also meant that only one bust and the name of the ruler appeared on the coins, which was associated with the transfer of the principle of primogeniture to succession in the dynasty. From 1551, the sons of the imprisoned John Frederick minted coins in Saalfeld with the portrait of Emperor Charles V. The final division of coins between the two Saxon lines introduced by Maurice perpetuated the division of coinage [32].

The patina investigation could identify some aspects that cannot be found in historical records, being more related to chance and human behavior. The physicochemical results indicate its prolonged contact with human skin during exposure to the outdoor atmosphere, suggesting it was received and kept as jewelry by a nomadic population. This hypothesis, substantiated by the patina composition, is also very plausible, explaining its long journey from XVIth-century Saxony to XXIst-century Transylvania. Most of the Romanian finds of Saxon coins are related to the hoards [72] that were accumulated like the Glogow Hoard, and there are fewer pierced random finds. These coins arrived in the Romanian area as a consequence of commercial activities, customs and taxes, being well represented by the issues of Upper Saxony [73]. A quarter thaler like the one found in the Glogow Hoard was reported in the Părău Hoard found in Transylvania [74].

On the other hand, John Frederick’s quarter thaler has a circulation path similar to the traders and rich people arriving in Glogow, where it was hoarded along with very good silver coins issued by other countries, like Czech and Moravian issues [75] or Transylvanian issues [76]. It was very well preserved without significant wearing marks, suggesting that it was mostly kept in coin boxes, not in the usual moneybags.

The rarity of these two Saxon quarter thalers issued in 1544 makes it difficult to examine a larger sample size, which represents the main limitation of the present study. A key goal for advancing this research in the future is to identify similar coins in other museum collections that are available for nondestructive analysis.

## 5. Conclusions

The two Saxon quarter thalers dated to 1544 analyzed in this study represent two iconographic variants associated with the coinage of Elector John Frederick I and Duke Maurice of Saxony. The numismatic characteristics of the coins correspond well with the historical organization of Saxon minting described in the literature, in which the Ernestine and Albertine branches of the Wettin dynasty shared mining revenues and participated in the joint issue of silver coinage. The differences observed in coin design—particularly the presence of four shields on the coin attributed to John Frederick and three shields on the coin associated with Maurice—are consistent with known typological variants recorded in Saxon numismatic studies. These features reflect the political and territorial symbolism commonly employed in the coinage of the period.

Nondestructive investigations effectuated with XRD, SEM-EDX and XRF reveal that both investigated coins have almost the same silver content on their surface: Maurice’s coin has 95.69 wt.%, and John Frederick’s coin has slightly less silver, at about 95.4. This significantly increased value is explained by the corrosion mechanisms that affect the thin copper lamellas within the Ag-Cu eutectic grains, which are partly removed from the coin’s surface by cleaning procedures. These finds are also corroborated by the primary sources, indicating that John Frederick minted coins of a slightly lower standard than his cousin Maurice.

The mild abrasion of small areas on the less evident sides of the John Frederick’s quarter thaler allows the proper investigation of bulk composition, which states that the silver amount is about 91 wt.%, in good agreement with the historical records.

The detected trace of lead can be explained by contemporary metallurgical practices, particularly the cupellation process used in the refinement of silver extracted from galena ores. Small residual quantities of lead were technologically unavoidable and therefore represent a typical by-product of sixteenth-century silver production rather than evidence of intentional debasement.

The physicochemical investigation of patina supports the hypothesis that the pierced John Frederick’s quarter thaler was used as jewelry, most likely worn by the nomadic population, with a tumultuous journey from the mint gate in 1544 to the Transylvanian random find in the XXI century.

The analytical results thus seem largely consistent with the technological and monetary standards documented in historical and numismatic sources. However, given that the present study examines only two specimens, the observations should be treated primarily as a case study illustrating the metallurgical characteristics of individual coins rather than as a comprehensive reconstruction of Saxon minting practice. Nevertheless, the analytical data obtained here demonstrate how archaeometric methods can complement traditional numismatic analysis and contribute to a better understanding of the material properties of early modern coinage.

## Figures and Tables

**Table 1:** XRD data for the patina layer correlated with mineralogical microscopy observation.

Chemical Formula	AgO	Ag_2_O_3_	Ag_2_S	CuO	Cu_2_O
Mineral name	argentic oxide	silver rust	argentite	tenorite	cuprite
Polarized light color	grey-black	yellow	dark-black	black	brown-reddish
Amount, wt.%	27	24	16	18	15
Crystallite size, nm	95	74	53	84	66
Cluster size, µm	10–50	5–25	5–22	20–40	15–35

**Table 2:** Coin denomination, weight and silver title valid in 1544.

Denomination	Guldengroschen	½ Guldengroschen	¼ Guldengroschen	Zingroschen (12 Pfennige)	Dreier (3 Pfennige)	Pfennige
Weight, g	29.23	14.62	7.31	2.66	1.19	0.4
Fineness, %	90.278	90.278	90.278	46.90	25.0	25.0

Note: Guldengroschen = thaler.

## Data Availability

The original contributions presented in this study are included in the article. Further inquiries can be directed to the corresponding author.

## Abbreviations

The following abbreviations are used in this manuscript:

AFM	Atomic Force Microscopy
BSE	Backscattered Electron
MOM	Mineralogical Optical Microscopy
SEI	Secondary electron Image
SEM	Scanning Electron Microscopy
EDS	Energy-Dispersive Spectroscopy
XRD	X-ray Diffraction
XRF	X-ray Fluorescence
